# Electronic cigarettes cause alteration in cardiac structure and function in diet-induced obese mice

**DOI:** 10.1371/journal.pone.0239671

**Published:** 2020-10-01

**Authors:** Kamrul M. Hasan, Theodore C. Friedman, Meher Parveen, Jorge Espinoza-Derout, Francisco Bautista, Mohammad M. Razipour, Xuesi M. Shao, Maria C. Jordan, Kenneth P. Roos, Sushil K. Mahata, Amiya P. Sinha-Hikim

**Affiliations:** 1 Division of Endocrinology, Metabolism and Molecular Medicine, Department of Internal Medicine, Charles R. Drew University, Los Angeles, CA, United States of America; 2 David Geffen School of Medicine at University of California, Los Angeles, CA, United States of America; 3 VA San Diego Health Care System and University of California, San Diego, CA, United States of America; Tel Aviv Sourasky Medical Center, ISRAEL

## Abstract

In spite of the widespread use of electronic cigarettes, also known as e-cigarettes, and the proposed adverse cardiac effects of nicotine, the detrimental effects of e-cigarettes on the heart are not well known. This study examines the detrimental effects of e-cigarettes with nicotine at doses that yield circulating nicotine and cotinine in the ranges similar to the levels found in habitual smokers, and a high fat diet (HFD) on cardiac structure and function in a commonly used model of diet-induced obesity (DIO). C57BL/6J mice on an HFD were exposed to e-cigarette in the presence (2.4% nicotine) or absence (0% nicotine) of nicotine and saline aerosol for 12 weeks. Echocardiographic data demonstrated a decrease in left ventricular (LV) fractional shortening, LV ejection fraction, and velocity of circumferential fiber shortening (VCF) in mice treated with e-cigarette (2.4% nicotine) compared to e-cigarette (0% nicotine) or saline exposed mice. Cardiomyocytes (CMs) of mice treated with e-cigarette (2.4% nicotine) exhibited LV abnormalities, including lipid accumulation (ventricular steatosis), myofibrillar derangement and destruction, and mitochondrial hypertrophy, as revealed by transmission electron microscopy. The detrimental effects of e-cigarettes (2.4% nicotine) on cardiac structure and function was accompanied by increased oxidative stress, plasma free fatty acid levels, CM apoptosis, and inactivation of AMP-activated protein kinase and activation of its downstream target, acetyl-CoA-carboxylase. Our results indicate profound adverse effects of e-cigarettes (2.4% nicotine) on the heart in obese mice and raise questions about the safety of the nicotine e-cigarettes use.

## Introduction

Smoking constitutes a major risk factor for cardiovascular disease (CVD), including myocardial infarction, sudden death, stroke, and peripheral vascular disease [1]. There is also a dose-response correlation between the smoking intensity (the number of cigarette smoked) and CVD morbidity and mortality [1]. Smoking also constitutes a risk factor for cardiomyopathy, a growing cause of morbidity and mortality [1]. What is frightening is that the usages of nicotine products such as transdermal patches, nicotine gum, and electronic cigarettes also known as e-cigarettes, in particular, are increasing [2–4]. As their popularity has increased, e-cigarettes have become the subject of a public health dispute as some experts welcome them as a pathway to the reduction or cessation of tobacco use, while opponents characterize them as a dangerous product that could undermine efforts to de-normalize smoking [5]. Emerging data show that e-cigarettes may also pose heart risk. A recent cross-sectional case-control study of habitual e-cigarette users (n = 23) and non-user control individuals (n = 19) showed increased cardiac sympathetic activity and oxidative stress, both associated with increased cardiovascular risk, in habitual e-cigarette users [6]. Recent studies also consistently reveal that most people who use e-cigarettes are so-called dual users, meaning that they use e-cigarettes as well as conventional cigarettes [7–9]. Dual use of cigarettes and e-cigarettes may lead to an increased risk of cardiopulmonary health [10, 11]. As e-cigarettes are a relatively new product, studies are needed to determine their long-term detrimental effects, some of which may be similar to the long-term detrimental effects of nicotine.

Recently, using Apolipoprotein null mice (*ApoE-/-*) on a western diet, a mouse model of nonalcoholic fatty liver disease [12, 13] and atherosclerosis [14], we further demonstrated the detrimental effects of e-cigarettes albeit at high dosages on the liver [15] as well as on the heart [16]. In the current study, we investigated some of the mechanisms underlying the detrimental effects of e-cigarettes, specifically at doses that deliver nicotine in the ranges similar to the clinically relevant concentrations (8–30 ng/mL) found in habitual e-cigarettes users [17, 18], and a high-fat diet (HFD), on cardiac structure and function in a mouse model of diet-induced obesity (DIO).

## Materials and methods

### Mice and tissue preparation

Adult male (10-week) old C57BL/6J mice (22–24 g BW) purchased from Taconic Farms (Germantown, NY, USA), were housed (2–3 per cage) in a standard animal facility under controlled temperature (22°C) and photoperiod (12-h light and 12-h dark cycle) with food and water *ad libitum*. Mice (n = 5) were fed an HFD with 60% of calories derived from fat (5.24 kcal/g; D12492; Research Diets, New Brunswick, NJ, USA). Animals were exposed to bluCig PLUS Classic tobacco e-cigarette containing 2.4% nicotine (purchased on the bluCig website) or saline aerosol daily for 12 hours (9:00 PM to 9:00 AM) for 12 weeks as previously described [16, 19]. These e-cigarettes are battery powered devices that use a resistive heating coil to aerosolize e-liquid or vehicle containing propylene glycol (PG), vegetable glycerin (VG), water, flavorings, and nicotine. According to the bluCig website [20], bluCig tanks contain nicotine, propylene glycol and vegetable glycerin, natural, and artificial flavors and distilled water, although there are no independent organizations that verify their content.

Our newly developed e-cigarettes aerosol exposure system can be programmed to deliver various doses of nicotine in rodents that yield plasma nicotine and cotinine levels in the ranges at very low, low, medium, and high [21]. Gold leaf tobacco (otherwise identical to Classic Tobacco) with 0% nicotine was used as an additional control to know if our findings are due to the nicotine in the e-cigarettes or to the vaporization of other non-nicotine e-cigarette ingredients.

Mice were returned to their home cages during the light phase of 12 hours (9 AM to 9 PM) and no aerosol was delivered. We used only male C57BL/6J mice since only male mice when fed an HFD develop visceral adiposity, hyperglycemia, insulin and leptin resistance, and metabolic syndrome [22, 23]. Mice were weighed weekly. Mice fasted overnight before euthanization with a lethal injection of sodium pentobarbital (200 mg/kg BW). We carefully removed, dissected ventricles from 5 mice in each experimental group. Portions of left ventricles were either fixed in 2.5% glutaraldehyde for high-resolution light and electron microscopy or 4% formalin for terminal deoxynucleotidyl transferase-mediated deoxyuridine triphosphate nick end labeling (TUNEL), and routine histological and immunohistochemical studies as described previously [24]. Five randomly selected left ventricular sections of ~5 μm in thickness and taken 50 μm apart per animal in each group were viewed to obtain the rate of CM apoptosis or for immunohistochemical analyses and computerized densitometry. Moreover, in order to minimize intra-sample variability, ventricular sections from the various experimental groups were processed for either TUNEL or immunolabeling for a given antibody in a single batch by using identical reagents. Altogether, five batches were processed.

The ventricle from additional groups of 5 mice were quickly removed snap-frozen in liquid nitrogen and stored -80°C for Western blot analysis. Animal handling and experimentation were in accordance with the recommendation of the current National Institutes of Health guidelines and were approved by the Charles R. Drew University School of Medicine and Science Institutional Animal Care and Use Committee (IACUC).

### Measurements of plasma nicotine, cotinine, and free fatty acid (FAA) levels

Plasma was collected at the time of sacrifice via cervical decapitation for determination of nicotine and cotinine levels at the UCSF Clinical Pharmacology Laboratory using LC-LC/MS [25]. The detection limit for nicotine is 2.5 ng/mL and for cotinine is 5 ng/m. Plasma FAA levels were measured by using Abcam’s Free Fatty Acid Assay kit according to manufacturer’s protocol (Cat. No. ab65341).

### Echocardiography

Echocardiography was done after the 12-week exposure period with e-cigarettes in the presence (2.4% nicotine) or absence (0% nicotine) of nicotine and saline aerosols at the Mouse Physiology Core Laboratory at UCLA Department of Physiology. To measure cardiac function, we employed a non-invasive ultrasound echocardiography under light isoflurane sedation (0.5% - 1.0%) to prevent movement and cardio-depression as described previously [26, 27]. Data were generated using a 2D-guided M-Mode & spectral Doppler imaging with a Siemens Acuson Sequoia Model C256 equipped with a 15L8 15 MHz probe (Siemens Medical Solutions, Malvern, CA). Heart dimension and function measurements were obtained via short axis M-Mode imaging. These included left ventricular (LV) chamber size, wall thickness, end-diastolic dimension (EDD), end-systolic dimension (ESD), LV fractional shortening (LV%FS), velocity of circumferential fiber shortening (VCF), and LV ejection fraction (LVEF). Aortic ejection time (Ao-ET) was obtained via long axis Doppler imaging. Early diastolic LV filling (E) and atrial systolic (A) values were obtained via apical view Doppler imaging of the mitral valve inflow. An observer who was unaware of the treatment assignment performed and analyzed the ultrasound measures.

### Transmission electron microscopy (TEM)

For electron microscopic studies, we used three glutaraldehyde-fixed left ventricles. Portions of three glutaraldehyde-fixed left ventricles were cut into small pieces, post-fixed in 1% osmium tetroxide, and embedded in Epon 812 as described previously [15, 16, 24] Thin sections from selected ventricles exhibiting pale gold interference color were cut with an LKB ultramicrotome, stained with uranyl acetate and lead citrate, and examined with a Hitachi electron microscope (Hitachi, Indianapolis, IN, USA).

### Cardiomyocyte (CM) apoptosis

Visualization of apoptotic cardiomyocyte was achieved in formalin-fixed, paraffin-embedded ventricular sections by the TUNEL technique [15, 24] using an ApopTag-peroxidase kit (Chemicon International, Inc., San Francisco, CA). Slides were counterstained with methyl green for detection of non-apoptotic nuclei. Enumeration of CM nuclei (both apoptotic and non-apoptotic) was determined within a reference area using an unbiased 2-dimension rule [28] as described previously [12, 24]. For each mouse, at least 50 grid fields were counted. The rate of apoptosis was expressed as the percentage of the TUNEL-positive nuclei per total nuclei (apoptotic and non-apoptotic) present within the reference area [12, 24].

### Measurements of oxidative stress

Measurements of oxidative stress was achieved by immunohistochemical analysis of 4-hydroxynonenal protein adducts (4-HNE), a biomarker of oxidative stress [29, 30], as described previously [12, 15, 24]. Formalin fixed, paraffin-embedded left ventricular sections were immunostained with mouse monoclonal 4-HNE antibody (1:100) from Oxis International Inc., Foster City, CA. Slides were counterstained with hematoxylin. We used a computerized densitometry employing the ImagePro Plus, version 5.1 software (Media Cybernetics, Silver Spring, MD) to quantify staining intensity as described previously [12, 24]. Results were further substantiated by Western blotting (see below) of ventricular 4-HNE expression in various groups.

### Western blotting

Western blotting was carried out using ventricular lysates as described previously [15, 24, 31] using a mouse monoclonal 4-HNE antibody (1: 1000) and rabbit polyclonal phospho-AMP-activated protein kinase (AMPK) (1:1000), total AMPK (1:1000), phospho-acetyl coenzyme-A-carboxylase (ACC) (1:1000), total ACC, and cleaved caspase 3 (1:1000) antibodies for overnight at 4°C with constant shaking. GAPDH was used for normalization of loading and was achieved using anti GAPDH antibody (1: 5000). We used Image J software to determine band intensities.

### Statistical analyses

We used SigmaStat 2.0 Program (Jandel Corporation, San Rafael, CA, USA) to provide statistical analyses. Data were expressed as mean ± SEM. Statistically significant difference among various treatment groups were defined using one-way Analysis of Variance (ANOVA), followed by the post-hoc (pairwise) comparisons using using Tukey’s or Tukey-Kramer’s tests. Differences were considered significant if p< 0.05.

## Results

### Body weight and plasma nicotine, cotinine and FAA levels

**Fig 1:**
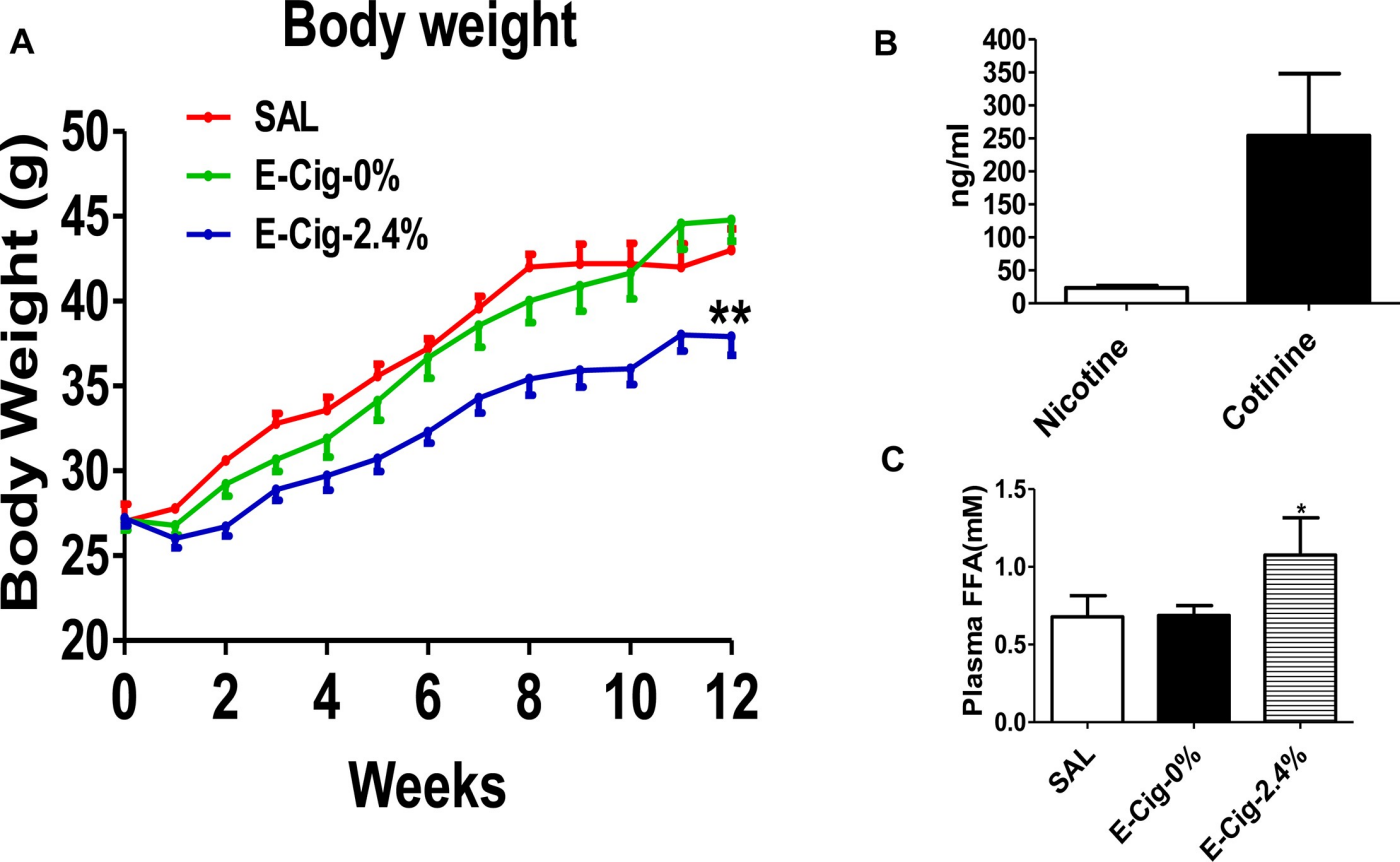
Body weight and plasma nicotine, cotinine, and FAA levels. (**A**) Body weight measured over 12 weeks in mice exposed to saline and e-cigarettes in the absence or presence of nicotine. Nicotine delivered through e-cigarettes (2.4% nicotine) significantly reduces body weight in mice on an HFD compared to HFD-fed mice exposed to saline or e-cigarette (0% nicotine) aerosol. There is no difference in body weight between mice on an HFD exposed to saline or e-cigarette (0% nicotine) aerosol. (**B**) Plasma nicotine and cotinine levels in mice exposed to e-cigarette. (**C**) Plasma FAA levels in e-cigarette and saline-exposed mice. Values are given as means ± SE of 5 mice per group. * P<0.05; ** P<0.01.

As expected from our previous studies [32, 33] with IP injections of nicotine, nicotine delivered through e-cigarettes (2.4% nicotine) significantly decreased body weight in HFD-fed mice compared to mice on an HFD exposed to saline or e-cigarette (0% nicotine) aerosol (**Fig 1A**). Body weight was essentially similar between mice on an HFD exposed to saline or e-cigarette (0% nicotine) aerosol. By 12 weeks of combined treatment with e-cigarette (2.4% nicotine) and HFD, mean body weight was decreased by 12% compared to mice exposed to saline or by 16% relative to mice exposed to e-cigarette (0% nicotine). After 12 weeks of e-cigarette (2.4% nicotine) treatment, plasma nicotine and cotinine levels were 23.4 ± 3.3 and 254.1 ± 42.1 (means ± SEM; ng/mL), respectively (**Fig 1B**). The observed levels of nicotine and cotinine appeared to be similar to the to the clinically relevant concentrations found in e-cigarettes users (8-30ng/mL) [17, 18] and habitual smokers with nicotine levels ranging from 10 ng/mL to 40 ng/mL and cotinine levels ranging from 100 ng/mL to 300 ng/mL [34, 35]. In contrast, plasma nicotine and cotinine levels were undetected in mice that received saline or e-cigarette (0% nicotine) exposure. Compared with HFD-fed exposed to saline or e-cigarette (0% nicotine) aerosols, mice on an HFD expose to e-cigarette (2.4% nicotine) exhibited a significant (P<0.05) increase in plasma FAA levels (**Fig 1C**).

### E-cigarette (2.4% nicotine) plus an HFD induces left ventricular ultrastructural defects in obese mice

**Fig 2:**
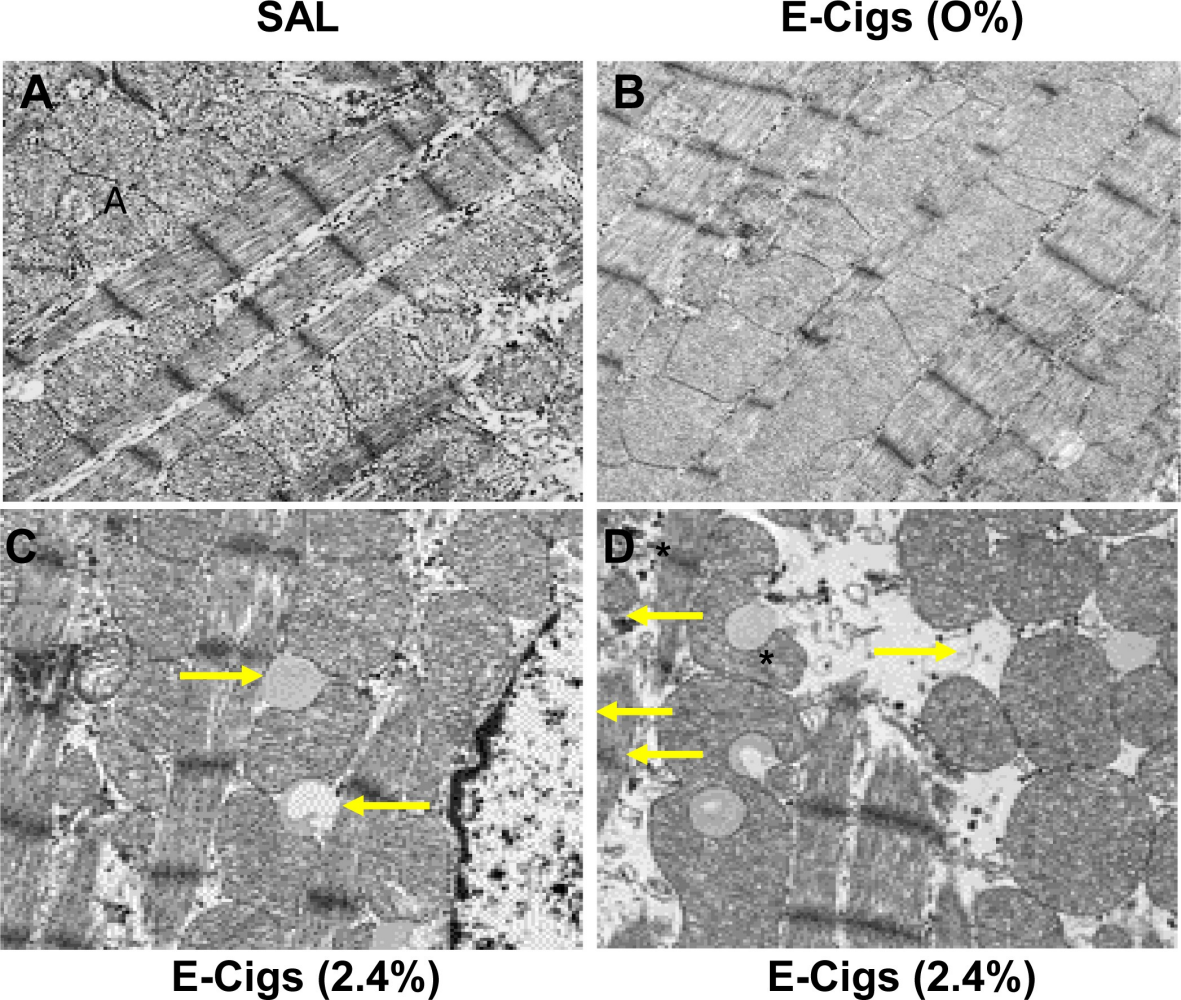
E-cigarette (2.4% nicotine) plus HFD induces left ventricular ultrastructural abnormalities in obese mice. (**A-D**) Representative TEM images of left ventricular myofibrillar architecture in various treatment groups. CMs from saline-(**A**) or e-cigarette (0% nicotine)-exposed (**B**) mice on an HFD shows normal myofibrillar architecture and sarcomeric pattern with normal nuclei and abundant mitochondria (m). In contrast, CMs from e-cigarette (2.4% nicotine) plus HFD exposed mice shows varying degrees of abnormalities (**C, D**), indicative of cardiomyopathy, including ventricular intramyocardial lipid accumulation (yellow arrow) or ventricular steatosis (**C, D**), myofibrillar derangement, thinning, and destruction (asterisks), and mitochondrial hypertrophy (**C, D**). Scale bar = 1μm.

We used TEM to assess left ventricular myofibrillar composition in various groups (**Fig 2A–2D**). CMs from saline- or e-cigarette (0% nicotine)-exposed mice on an HFD showed normal myofibrillar composition and sarcomeric arrangement accompanied by normal nuclei and abundant mitochondria (**Fig 2A and 2B**). However, CMs from e-cigarette (2.4% nicotine) plus HFD treated mice showed ultrastructural abnormalities (**Fig 2C and 2D**), indicative of cardiomyopathy [36, 37]. These included ventricular intramyocardial lipid accumulation or ventricular steatosis (**Fig 2C**), myofibrillar derangement, thinning, and destruction, and mitochondrial hypertrophy (**Fig 2C and 2D**).

### Echocardiography

To reveal functional changes induced by e-cigarettes (2.4% nicotine) and HFD, we performed echocardiography in HFD-fed mice treated with saline, e-cigarette (0% nicotine), or e-cigarette (2.4% nicotine) aerosol. LV dimensions measured by M-mode measurements exhibited no differences among saline, e-cigarette (0% nicotine), and e-cigarette (2.4% nicotine) exposed groups (**Table 1**). There was also no change in the heart rate among various experimental groups (**Table 1**). To determine the left ventricular systolic function, we measured LV%FS, LVEF, and VCF. All these parameters were significantly decreased in the HFD-fed mice exposed to e-cigarettes (2.4% nicotine) relative to those exposed with saline or e-cigarette without nicotine aerosol. No apparent changes in the LV diastolic functional parameters such as peak early diastolic (E), atrial filling velocity (A), and E/A ratio was noted in mice in various treatment groups. Taken together, these results indicate that exposure of nicotine delivered via e-cigarettes (2.4% nicotine) impairs ventricular systolic function. The absence of an effect of non-nicotine e-cigarettes (0% nicotine) shows that nicotine is critical for the observed systolic dysfunction induced by e-cigarette (2.4% nicotine).

**Table 1:** Echocardiography data after 12 weeks exposure to E-cigarette in the presence or absence of nicotine or saline aerosol.

Parameter	Saline: mean ± SD	E-Cigarettes-(0%): mean ± SD	E-Cigarettes (2.4%): mean ± SD
VST(mm)	0.43 ± 0.03	0.44 ± 0.06	0.44 ± 0.03
EDD(mm)	4.62 ± 0.08	4.58 ± 0.08	4.54 ± 0.19
PWT	0.44 ± 0.02	0.45 ± 0.04	0.45 ± 0.04
ESD	3.04 ± 0.29	3.02 ± 0.51	3.26 ± 0.17
Ao-ET(ms)	52.52 ± 4.36	49.1 ± 2.86	54.24 ± 6.42
HR	507.46 ± 39.23	544.83 ± 45.63	499.47 ± 93.27
LV mass	70.51 ± 5.48	72.81 ± 18.32	70.47 ± 1.43
LV% FS	34.2 ± 6.3	34.4 ± 6.2	28.2 ± 1.6*
LVEF	69.1 ± 8.3	69.2 ± 8.1	61.6 ± 3.5*
VCF	6.47 ± 0.71	6.91 ± 1.15	5.28 ± 0.88*
E	0.77 ± 0.2	0.79 ± 0.07	0.85 ± 0.19
A	0.37 ± 0.09	0.38 ± 0.06	0.38 ± 0.08
E/A	2.09 ± 0.24	2.1 ± 0.33	2.23 ± 0.3

Abbreviations: VST, ventricular septal thickness; PWT, posterior wall thickness; EDD, end-diastolic dimension; ESD, end systolic dimension; AoEt, aortic ejection time; HR, heart rate; LV%FS, left ventricle fractional shortening; VCF, velocity of circumferential fiber shortening; LVEF, left ventricle ejection fraction; LV Mass, left ventricle mass; E, early diastole (LV filling); A, atrial systole: H/B ratio, Heart/body ratio.

* P<0.05 (n = 5).

### E-cigarettes (2.4% nicotine) trigger CM apoptosis

**Fig 3:**
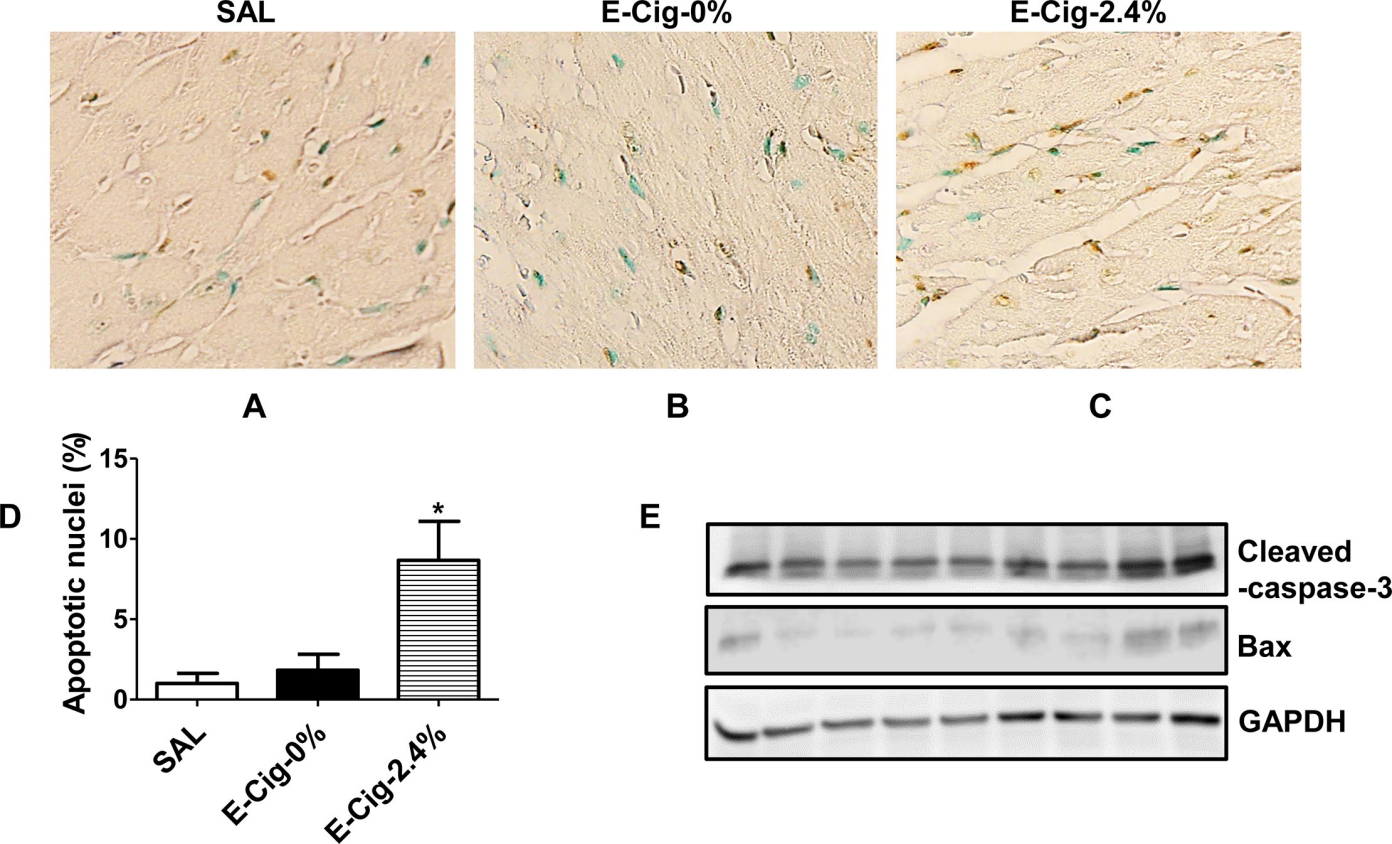
E-cigarettes (2.4% nicotine) trigger CM apoptosis. Compared to mice exposed to saline (**A**) or e-cigarette (0% nicotine) aerosol (**B**), where little or no apoptosis is detected, a significant increase in CM apoptosis (**C**) was detected in mice exposed to e-cigarettes (2.4% nicotine). Scale bar = 25 μm. (**D**) Quantitation of CM apoptosis. Values are given as means ± SE of 5 mice per group. * P<0.05. (**E**) Western blot analysis of active (cleaved) caspase 3.

Because CM apoptosis plays an important role for the development of cardiomyopathy and heart failure [38, 39], we next assessed the additive effects of e-cigarettes (2.4% nicotine) and HFD on CM apoptosis. Minimal CM apoptosis was noted in mice treated with saline or non-nicotine e-cigarette aerosol (**Fig 3A and 3B**). In contrast, e-cigarette (2.4% nicotine) exposed ventricles had significantly increased rate of ventricular CM apoptosis over the values measured in saline (by 8.6-fold) or non-nicotine e-cigarette exposed (by 4.8-fold) mice (**Fig 3A and 3B**). As an additional measure of CM apoptosis, we carried out western blot analysis of active (cleaved) caspase 3. Densitometric analysis showed a significant increase in the ventricular expression of active caspase 3 in HFD-fed mice treated with e-cigarette (2.4% nicotine) relative to HFD-fed mice exposed to saline or e-cigarette (0% nicotine) aerosol (**Fig 3C and 3D**).

### E-cigarettes (2.4% nicotine) plus HFD inactivates AMPK and triggers oxidative stress

**Fig 4:**
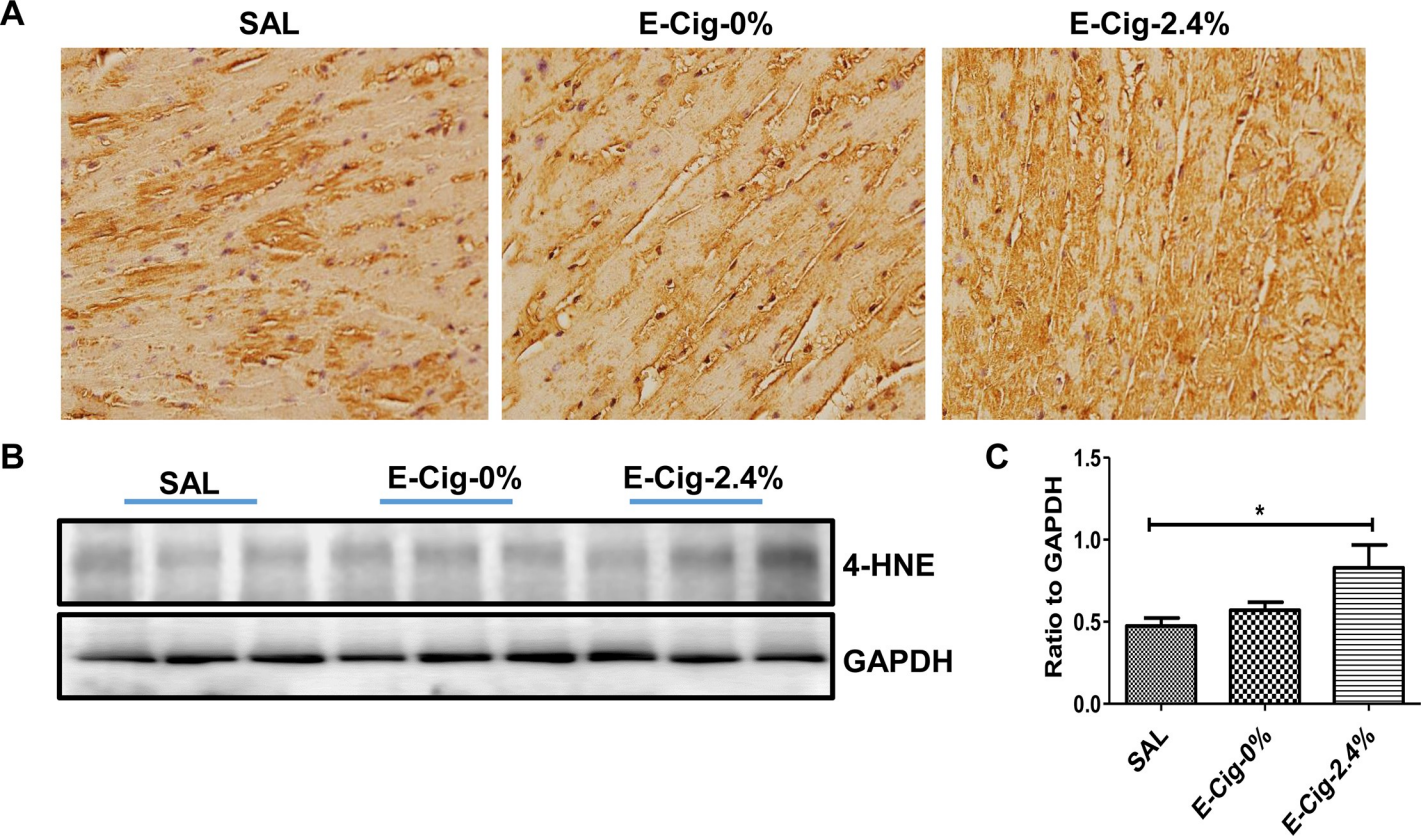
E-cigarettes (2.4% nicotine) plus HFD inactivates AMPK. (**A**) Western blot analysis of ventricular expression of phospho(p)-AMPK, total AMPK, phospho (p)-ACC, total ACC shows that mice on an HFD exposed to e-cigarette have markedly decreased p-AMPK and p-ACC levels compared with mice on an HFD exposed to saline or e-cigarette (0% nicotine) aerosol. (**B, C**) Quantitation of band intensities. GAPDH in the immunoblot is shown as a loading control. P<0.05. Values are given as means ± SE of 5 mice per group. * P<0.05.

AMPK, a key player involved in myocardial metabolic processes [40], plays an important role in regulating CM apoptosis [41]. To investigate whether e-cigarette (2.4% nicotine) plus HFD-induced CM apoptosis is accompanied by inactivation of AMPK, we tested the phosphorylation state of AMPK in ventricular lysates by western blotting. Compared to mice on an HFD exposed to saline or e-cigarette (0% nicotine) aerosol, combined treatment with e-cigarette (2.4% nicotine) and HFD resulted in a significant reduction in ventricular phospho-AMPK levels (**Fig 4A and 4B**). The net effect of inactivation of AMPK in e-cigarette (2.4% nicotine) exposed ventricles was no ventricular phospho-ACC detected in HFD plus e-cigarette (2.4% nicotine)-treated groups (**Fig 4A and 4C**).

**Fig 5:**
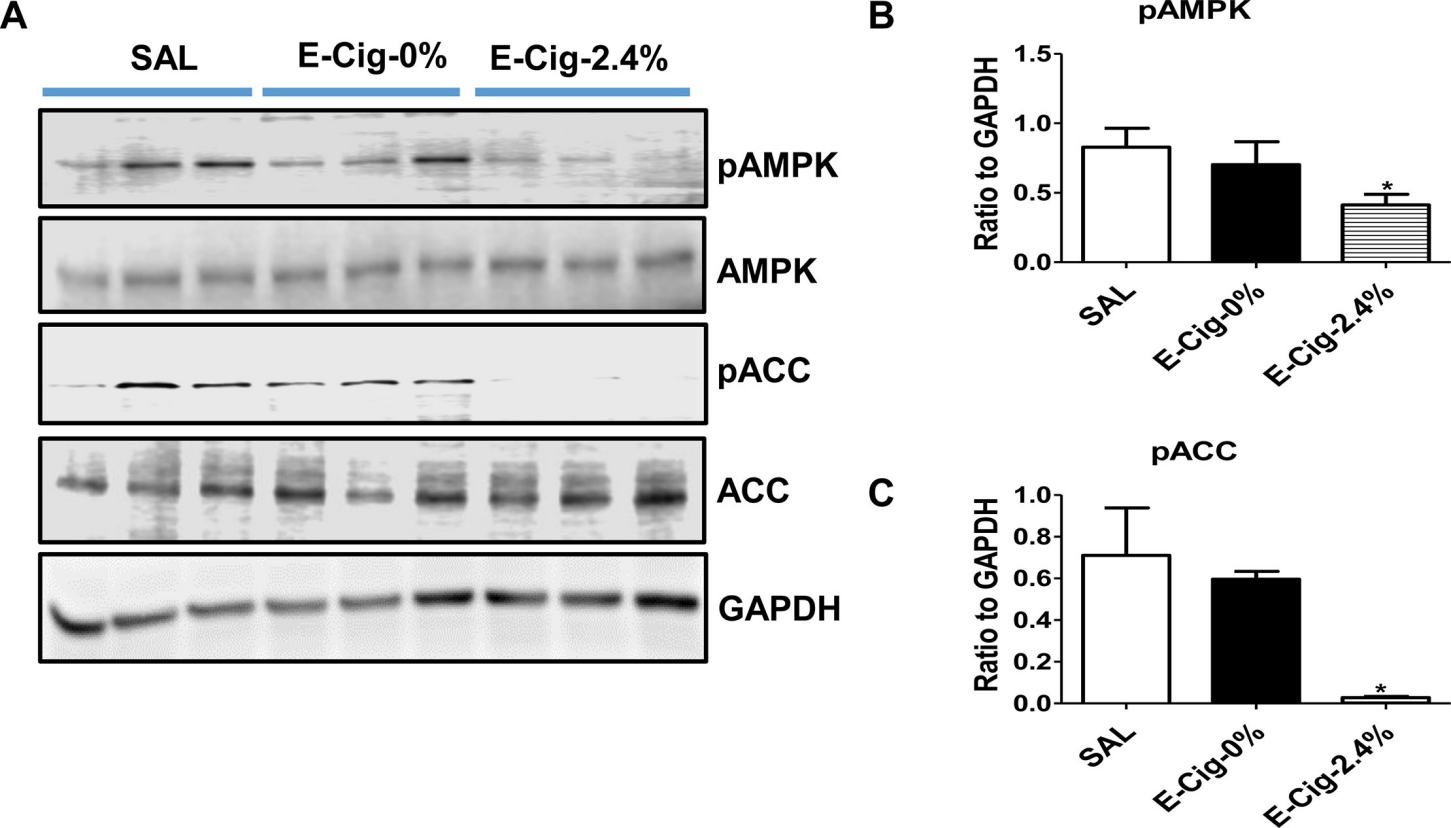
E-cigarettes (2.4% nicotine) plus HFD triggers oxidative stress. Immunohistochemical analysis of ventricular sections show increased expression of 4-HNE in mice exposed to e-cigarette (2.4% nicotine) compared with saline- or e-cigarette (0% nicotine)-treated mice (**A).** Western blot analysis shows increased 4-HNE levels in mice exposed to e-cigarette (**B**). (**C**) Quantitation of band intensities. Data for p-AMPK and p-ACC were normalized to GAPDH. P<0.05. Values are given as means ± SE of 5 mice per group. * P<0.05.

To test whether e-cigarette plus HFD causes greater oxidative stress, we compared in vivo ventricular expression of a lipid peroxidation product 4-HNE, a biomarker of oxidative stress [29, 30] in various treatment groups. Compared with saline or e-cigarette (0% nicotine)-treated mice, mice exposed to e-cigarette (2.4% nicotine) had higher 4-HNE immunoreactivity (**Fig 5A**). We also performed western blot analysis of 4-HNE. Compared with mice on a HFD exposed to saline or e-cigarette (0% nicotine) aerosol, HFD fed mice exposed to e-cigarette (2.4% nicotine) had significantly higher 4-HNE expression (**Fig 5B and 5C**).

## Discussion

In an earlier study in C57BL/6J mice, we demonstrated that nicotine (administered intraperitoneally) when combined with an HFD triggers CM apoptosis through the generation of oxidative stress and inactivation of AMPK together with activation of the caspase 2-mediated intrinsic apoptotic signaling [24]. More recently, using *ApoE-/-* mice on a western diet, a mouse model of nonalcoholic fatty levers disease [12, 13] and atherosclerosis [14], we further demonstrated that the detrimental effects of nicotine delivered through e-cigarettes (2.4% nicotine) at high dosages that led to equivalent serum cotinine levels found in heavy smokers [42] on the heart [16]. We noted in that study that *ApoE-/-* mice on a WD exposed to nicotine e-cigarettes (2.4% nicotine) had decreased LV%FS, LVEF, and VCF coupled with LV ultrastructural abnormalities indicative of cardiomyopathy [36, 37] in comparison with e-cigarette (0% nicotine) or saline-aerosol exposed mice.

The current study is unique in that it employs a commonly used model of DIO [22, 23, 43] and examines the harmful effects of two common lifestyle factors, nicotine (delivered through e-cigarettes) specifically at dose levels that deliver plasma nicotine and cotinine levels similar to the clinically relevant concentrations found in habitual smokers and e-cigarette users, and HFD, on cardiac structure and function. The results of the present study confirm and extend our earlier report [16] by demonstrating that: 1) nicotine and HFD impairs ventricular systolic function and causes LV steatosis and ultrastructural abnormalities indicative of cardiomyopathy and cardiac dysfunction and 2) the detrimental effects of e-cigarettes (2.4% nicotine) and HFD on cardiac structure and function are mediated by multiple mechanisms involving induction of oxidative stress and CM apoptosis, excess FFA delivery (possibly through adipose tissue lipolysis), and inactivation of AMPK and activation of its downstream target ACC. These findings underscore the adverse effects of nicotinic e-cigarettes on the heart. Based on the transmitral E & A flow velocity pattern, diastolic function did not appear to change significantly. In human subjects the assessment of diastolic function is complex, and requires advanced techniques such as tissue Doppler, E/e', pulmonary venous flow and additional parameters, which were not available in the current study.

The lack of an effect of e-cigarettes in the absence of nicotine (0% nicotine) shows that nicotine is necessary for cardiac abnormalities induced by e-cigarette (2.4% nicotine). In this context, it is worth noting that even short-term (14 days) e-cigarette exposure while had no acute effect on cardiac contractile function or tissue fibrosis, it did increase angiogenesis in C57BL/6J mice [44]. A recent study has also indicated the chronic (4h/day, 5days/wk, for 8 months) e-cigarette exposure accelerated arterial stiffness and impairs aortic endothelial function, both associated with cardiovascular risks in C57BL/6J mice [45]. The results of these studies, together with our current findings of additive effects of e-cigarette (2.4% nicotine) and HFD on cardiac function, indicate that e-cigarettes (2.4% nicotine) alone may have potential cardiovascular risk, which can be exacerbated by combining with HFD. However, we cannot rule out the possibilities that other substances in the e-cigarettes other than nicotine may have some detrimental cardiac effects [46].

In the heart, AMPK has been recognized as a key modulator of energy metabolism where it regulates fatty acid and glucose transport/utilization during both physiological and pathological states and is an essential survival component against diverse CMs stress [47, 48] Consistent with a pivotal role of AMPK in the heart, here we show that combined treatment with e-cigarette and HFD that caused cardiac structural and functional abnormalities, also caused inhibition of AMPK. ACC is phosphorylated and inhibited by AMPK [49]. The net effect of AMPK inactivation is decreased phosphorylation and inhibition of ACC, leading to cardiac steatosis, possibly through decreased fatty acid oxidation in the heart [49]. This is also consistent with our earlier reports indicating that parenterally administered nicotine when combined with an HFD causes inhibition of AMPK in the liver [32], skeletal muscle [33], and in the heart [24]. Additional support of this notion comes from the findings that the reduction of AMPK β1 and β2 isoforms in cardiac muscle lead to significant reduction in phospho-AMPK and phospho-ACC and causes reduction in LV%FS, LVEF, and cardiac output, demonstrating that inhibition of AMPK is directly associated with cardiac systolic dysfunction [50]. AMPK-α2 deficiency also exacerbates pressure-overload-induced LV hypertrophy and dysfunction in mice [51]. It is worth noting that deficiency in adiponectin through inhibition of AMPK provokes cigarette smoke-induced cardiomyopathy but can be rescued by AICAR-mediated AMPK activation [52].

AMPK also plays an important role in regulating CM apoptosis [41, 47, 49, 53], which has been implicated as a potential mechanism in the development of cardiomyopathy and heart failure [38, 39] Consistent with a pivotal role of AMPK in myocardial cell apoptosis, here we show that nicotine e-cigarettes (2.4% nicotine) plus an HFD triggers CM apoptosis. This is in agreement with our earlier reports that nicotine, administered intraperitoneally, when combined with an HFD causes inhibition of AMPK in the heart and triggers CM apoptosis [24].

Oxidative stress has been implicated in apoptotic signaling in various cell types, including CMs [12, 53, 54]. Indeed, in the present study, we found that combined treatment with e-cigarette (2.4% nicotine) plus an HFD generated a greater oxidative stress, as evidenced by increase ventricular 4-HNE expression compared to e-cigarette (0% nicotine) or saline-exposed mice. Of further interest, emerging evidence suggests that inhibition of AMPK can also elevate oxidative stress in a variety of cell systems, including CMs [12, 53–56]. Thus, it is possible that inhibition of AMPK could increase the susceptibility of CMs to apoptosis in the nicotine e-cigarette (2.4% nicotine) plus HFD-exposed groups. One possible mechanism by which oxidative stress can induce CM apoptosis in response to HFD plus nicotine is through stimulation of intrinsic pathway signaling, which constitutes a critical component of apoptotic signaling in CMs [24].

Although our study has important implications in the rapidly expanding field of defining e-cigarette-induced toxicity in various organs, it has some limitations. One potential limitation is that our echo machine does not have tissue Doppler capability nor the capacity to calculate the E/e’ value and we have now acknowledged that value would provide additional information regarding diastolic dysfunction. Still, the E and A values obtained here provide useful index of diastolic function and are accurate. Mice are not humans and present many challenges for ultrasonic imaging. Most importantly, they have very small hearts and high heart rates which lead to short filling times. Thus, some criteria typical in humans do not necessarily apply to mice. It is very rare to see mice go into a clear heart failure except under extreme pathological circumstances or with aging. However, ventricular systolic dysfunction as seen in this study is common. The E and A values obtained are well validated by the UCLA physiology core lab here are very consistent with previous data in a wide variety of other mouse models. When they change, it is obvious and significant. In past studies on other mouse models, when the E/A ratio indicated diastolic dysfunction, we also saw significant fibrosis. Though we had structural defects and apoptosis, in this study there was no significant fibrosis. Unlike in humans, diastolic dysfunction does not necessarily precede systolic dysfunction in mice. An additional limitation of our study is that we used only males. There is growing evidence that female mice on a HFD also develop obesity related insulin resistance and glucose intolerance, and NAFLD [57, 58]. Thus, it is possible that the observed detrimental effects of e-cigarettes on the heart in HFD-fed male mice could also be seen in female mice on an HFD. Clearly, this merits further investigation. We also acknowledge that our study does not include a normal diet group and mice treated with nicotine through another administration route as controls. In this context it is worth noting here that in our earlier studies, we have demonstrated that intraperitoneal injections of nicotine when combined with a normal diet has no detrimental effects on various organs, including liver [32, 59], gastrocnemius muscle [33], and heart [24]. More specifically, in the heart study, we have found that nicotine only when combined with an HFD but not with a normal diet increases cardiac oxidative stress, inactivates AMPK, and causes CM apoptosis. Available evidence also suggests that nicotine delivered via mini osmotic pump can exacerbate angiotensin II-induced cardiovascular remodeling in a mouse model of systemic hypertension [2]. Data reported in the present study also demonstrate no adverse cardiac effects of e-cigarettes in the absence of nicotine (0% nicotine). At present, we also can not rule out the possibility that the observed detrimental effects of e-cigarettes that are obvious in this high nicotine dose might not be so obvious if given in lower dosages. Taken together, these results suggest that nicotine is most likely responsible for the observed cardiac effects. However, further work will be needed to understand if nicotine delivered by another route, such as transdermal patch, has similar effects.

In summary, our data show profound adverse effects of e-cigarette (2.4% nicotine) and HFD on cardiac structure and function. These detrimental effects on cardiac structure and function are mediated by multiple mechanisms involving induction of oxidative stress and CM apoptosis, excess FFA delivery (possibly through adipose tissue lipolysis), and inactivation of AMPK and activation of its downstream target ACC. Although further mechanistic studies are needed, the data provide an in vivo demonstration of profound adverse effects of e-cigarettes (2.4% nicotine) on the heart in obese mice. Our data, therefore, question the popular belief that nicotine e-cigarettes are safe and caution should be used both by consumers that use them and regulatory bodies that regulate them.

## Supporting information

S1 Raw images Uncropped wb data corrected. (PPTX)

## References

[1] Saleheen D, Zhao W, Rasheed A. Epidemiology and public health policy of tobacco use and cardiovascular disorders in low- and middle-income countries. Arterioscler Thromb Vasc Biol. 2014;34(9):1811–9. 10.1161/ATVBAHA.114.303826 . 25035346

[2] Colombo ES, Davis J, Makvandi M, Aragon M, Lucas SN, Paffett ML, et al Effects of nicotine on cardiovascular remodeling in a mouse model of systemic hypertension. Cardiovasc Toxicol. 2013;13(4):364–9. 10.1007/s12012-013-9217-z 23959951 PMC4070620

[3] Grando SA. Connections of nicotine to cancer. Nat Rev Cancer. 2014;14(6):419–29. 10.1038/nrc3725 . 24827506

[4] Hiemstra PS, Bals R. Basic science of electronic cigarettes: assessment in cell culture and in vivo models. Respir Res. 2016;17(1):127 Epub 2016/10/09. 10.1186/s12931-016-0447-z 27717371 PMC5055681

[5] Fairchild AL, Bayer R, Colgrove J. The renormalization of smoking? E-cigarettes and the tobacco "endgame". N Engl J Med. 2014;370(4):293–5. 10.1056/NEJMp1313940 . 24350902

[6] Moheimani RS, Bhetraratana M, Yin F, Peters KM, Gornbein J, Araujo JA, et al Increased Cardiac Sympathetic Activity and Oxidative Stress in Habitual Electronic Cigarette Users: Implications for Cardiovascular Risk. JAMA Cardiol. 2017;2(3):278–84. 10.1001/jamacardio.2016.5303 . 28146259 PMC5626008

[7] Pearson JL, Richardson A, Niaura RS, Vallone DM, Abrams DB. e-Cigarette awareness, use, and harm perceptions in US adults. Am J Public Health. 2012;102(9):1758–66. 10.2105/AJPH.2011.300526 22813087 PMC3474361

[8] Regan AK, Promoff G, Dube SR, Arrazola R. Electronic nicotine delivery systems: adult use and awareness of the 'e-cigarette' in the USA. Tob Control. 2013;22(1):19–23. 10.1136/tobaccocontrol-2011-050044 . 22034071

[9] Vickerman KA, Carpenter KM, Altman T, Nash CM, Zbikowski SM. Use of electronic cigarettes among state tobacco cessation quitline callers. Nicotine Tob Res. 2013;15(10):1787–91. 10.1093/ntr/ntt061 . 23658395

[10] Ma JZ, Hart JL, Walker KL, Giachello AL, Groom A, Landry RL, et al Perceived health risks of electronic nicotine delivery systems (ENDS) users: The role of cigarette smoking status. Addict Behav. 2019;91:156–63. Epub 2018/11/14. 10.1016/j.addbeh.2018.10.044 30420103 PMC6358486

[11] Wang JB, Olgin JE, Nah G, Vittinghoff E, Cataldo JK, Pletcher MJ, et al Cigarette and e-cigarette dual use and risk of cardiopulmonary symptoms in the Health eHeart Study. PLoS One. 2018;13(7):e0198681 Epub 2018/07/26. 10.1371/journal.pone.0198681 30044773 PMC6059385

[12] Sinha-Hikim I, Shen R, Nzenwa I, Gelfand R, Mahata SK, Sinha-Hikim AP. Minocycline suppresses oxidative stress and attenuates fetal cardiac myocyte apoptosis triggered by in utero cocaine exposure. Apoptosis. 2011;16(6):563–73. 10.1007/s10495-011-0590-4 21424555 PMC4701037

[13] Schierwagen R, Maybuchen L, Zimmer S, Hittatiya K, Back C, Klein S, et al Seven weeks of Western diet in apolipoprotein-E-deficient mice induce metabolic syndrome and non-alcoholic steatohepatitis with liver fibrosis. Sci Rep. 2015;5:12931 Epub 2015/08/12. 10.1038/srep12931 26263022 PMC4531783

[14] Pendse AA, Arbones-Mainar JM, Johnson LA, Altenburg MK, Maeda N. Apolipoprotein E knock-out and knock-in mice: atherosclerosis, metabolic syndrome, and beyond. J Lipid Res. 2009;50 Suppl:S178–82. Epub 2008/12/09. 10.1194/jlr.R800070-JLR200 19060252 PMC2674752

[15] Hasan KM, Friedman TC, Shao X, Parveen M, Sims C, Lee DL, et al E-cigarettes and Western Diet: Important Metabolic Risk Factors for Hepatic Diseases. Hepatology. 2019 10.1002/hep.30512 . 30664268 PMC6636679

[16] Espinoza-Derout J, Hasan KM, Shao XM, Jordan MC, Sims C, Lee DL, et al Chronic Intermittent Electronic Cigarette Exposure Induces Cardiac Dysfunction and Atherosclerosis in Apolipoprotein E (ApoE) Knockout Mice. Am J Physiol Heart Circ Physiol. 2019 Epub 2019/06/08. 10.1152/ajpheart.00738.2018 . 31172811 PMC6732484

[17] Farsalinos KE, Tsiapras D, Kyrzopoulos S, Savvopoulou M, Voudris V. Acute effects of using an electronic nicotine-delivery device (electronic cigarette) on myocardial function: comparison with the effects of regular cigarettes. BMC Cardiovasc Disord. 2014;14:78 10.1186/1471-2261-14-78 24958250 PMC4077146

[18] St Helen G, Havel C, Dempsey DA, Jacob P 3rd, Benowitz NL. Nicotine delivery, retention and pharmacokinetics from various electronic cigarettes. Addiction. 2016;111(3):535–44. Epub 2015/10/03. 10.1111/add.13183 26430813 PMC4749433

[19] Hasan KM, Friedman TC, Shao X, Parveen M, Sims C, Lee DL, et al E-cigarettes and Western Diet: Important Metabolic Risk Factors for Hepatic Diseases. Hepatology. 2019;69(6):2442–54. 10.1002/hep.30512 . 30664268 PMC6636679

[20] [February 10, 2018]. Available from: https://www.blu.com/en/US/flavors/blu-plus-tank-classic-tobacco-us.html.

[21] Shao XM, Lopez B, Nathan D, Wilson J, Bankole E, Tumoyan H, et al A mouse model for chronic intermittent electronic cigarette exposure exhibits nicotine pharmacokinetics resembling human vapers. J Neurosci Methods. 2019;326:108376 Epub 2019/07/31. 10.1016/j.jneumeth.2019.108376 31361999 PMC6717674

[22] Collins S, Martin TL, Surwit RS, Robidoux J. Genetic vulnerability to diet-induced obesity in the C57BL/6J mouse: physiological and molecular characteristics. Physiol Behav. 2004;81(2):243–8. 10.1016/j.physbeh.2004.02.006 . 15159170

[23] de Meijer VE, Le HD, Meisel JA, Akhavan Sharif MR, Pan A, Nose V, et al Dietary fat intake promotes the development of hepatic steatosis independently from excess caloric consumption in a murine model. Metabolism. 2010;59(8):1092–105. 10.1016/j.metabol.2009.11.006 20060143 PMC3361716

[24] Sinha-Hikim I, Friedman TC, Falz M, Chalfant V, Hasan MK, Espinoza-Derout J, et al Nicotine plus a high-fat diet triggers cardiomyocyte apoptosis. Cell Tissue Res. 2017;368(1):159–70. Epub 2016/12/06. 10.1007/s00441-016-2536-1 . 27917437 PMC5813800

[25] Jacob P 3rd, Yu L, Duan M, Ramos L, Yturralde O, Benowitz NL. Determination of the nicotine metabolites cotinine and trans-3'-hydroxycotinine in biologic fluids of smokers and non-smokers using liquid chromatography-tandem mass spectrometry: biomarkers for tobacco smoke exposure and for phenotyping cytochrome P450 2A6 activity. J Chromatogr B Analyt Technol Biomed Life Sci. 2011;879(3–4):267–76. 10.1016/j.jchromb.2010.12.012 21208832 PMC3050598

[26] Parvatiyar MS, Marshall JL, Nguyen RT, Jordan MC, Richardson VA, Roos KP, et al Sarcospan Regulates Cardiac Isoproterenol Response and Prevents Duchenne Muscular Dystrophy-Associated Cardiomyopathy. J Am Heart Assoc. 2015;4(12). 10.1161/JAHA.115.002481 26702077 PMC4845268

[27] Roos KP, Jordan MC, Fishbein MC, Ritter MR, Friedlander M, Chang HC, et al Hypertrophy and heart failure in mice overexpressing the cardiac sodium-calcium exchanger. J Card Fail. 2007;13(4):318–29. Epub 2007/05/23. 10.1016/j.cardfail.2007.01.004 17517353 PMC2017112

[28] Cruz-Orive LM, Weibel ER. Recent stereological methods for cell biology: a brief survey. Am J Physiol. 1990;258(4 Pt 1):L148–56. 10.1152/ajplung.1990.258.4.L148 . 2185653

[29] Kohen R, Nyska A. Oxidation of biological systems: oxidative stress phenomena, antioxidants, redox reactions, and methods for their quantification. Toxicol Pathol. 2002;30(6):620–50. 10.1080/01926230290166724 . 12512863

[30] Tam NN, Gao Y, Leung YK, Ho SM. Androgenic regulation of oxidative stress in the rat prostate: involvement of NAD(P)H oxidases and antioxidant defense machinery during prostatic involution and regrowth. Am J Pathol. 2003;163(6):2513–22. 10.1016/S0002-9440(10)63606-1 14633623 PMC1892368

[31] Hasan MK, Friedman TC, Sims C, Lee DL, Espinoza-Derout J, Ume A, et al alpha7-Nicotinic Acetylcholine Receptor Agonist Ameliorates Nicotine Plus High-Fat Diet-Induced Hepatic Steatosis in Male Mice by Inhibiting Oxidative Stress and Stimulating AMPK Signaling. Endocrinology. 2018;159(2):931–44. Epub 2017/12/23. 10.1210/en.2017-00594 29272360 PMC5776480

[32] Friedman TC, Sinha-Hikim I, Parveen M, Najjar SM, Liu Y, Mangubat M, et al Additive effects of nicotine and high-fat diet on hepatic steatosis in male mice. Endocrinology. 2012;153(12):5809–20. Epub 2012/10/25. 10.1210/en.2012-1750 23093702 PMC3512067

[33] Sinha-Hikim I, Friedman TC, Shin CS, Lee D, Ivey R, Sinha-Hikim AP. Nicotine in combination with a high-fat diet causes intramyocellular mitochondrial abnormalities in male mice. Endocrinology. 2014;155(3):865–72. 10.1210/en.2013-1795 24424058 PMC3929732

[34] Benowitz N, Dempsey D. Pharmacotherapy for smoking cessation during pregnancy. Nicotine Tob Res. 2004;6 Suppl 2:S189–202. 10.1080/14622200410001669169 . 15203821

[35] Hukkanen J, Dempsey D, Jacob P 3rd, Benowitz NL. Effect of pregnancy on a measure of FMO3 activity. Br J Clin Pharmacol. 2005;60(2):224–6. 10.1111/j.1365-2125.2005.02406.x 16042678 PMC1884929

[36] Rapti K, Diokmetzidou A, Kloukina I, Milner DJ, Varela A, Davos CH, et al Opposite effects of catalase and MnSOD ectopic expression on stress induced defects and mortality in the desmin deficient cardiomyopathy model. Free Radic Biol Med. 2017;110:206–18. 10.1016/j.freeradbiomed.2017.06.010 . 28629836

[37] Wang Y, Herron AJ, Worman HJ. Pathology and nuclear abnormalities in hearts of transgenic mice expressing M371K lamin A encoded by an LMNA mutation causing Emery-Dreifuss muscular dystrophy. Hum Mol Genet. 2006;15(16):2479–89. 10.1093/hmg/ddl170 . 16825283

[38] Mandl A, Huong Pham L, Toth K, Zambetti G, Erhardt P. Puma deletion delays cardiac dysfunction in murine heart failure models through attenuation of apoptosis. Circulation. 2011;124(1):31–9. 10.1161/CIRCULATIONAHA.110.988303 . 21670227

[39] Sun Y, Yao X, Zhang QJ, Zhu M, Liu ZP, Ci B, et al Beclin-1-Dependent Autophagy Protects the Heart During Sepsis. Circulation. 2018;138(20):2247–62. 10.1161/CIRCULATIONAHA.117.032821 29853517 PMC6274625

[40] Kim TT, Dyck JR. Is AMPK the savior of the failing heart? Trends Endocrinol Metab. 2015;26(1):40–8. 10.1016/j.tem.2014.11.001 . 25439672

[41] Zhuo XZ, Wu Y, Ni YJ, Liu JH, Gong M, Wang XH, et al Isoproterenol instigates cardiomyocyte apoptosis and heart failure via AMPK inactivation-mediated endoplasmic reticulum stress. Apoptosis. 2013;18(7):800–10. 10.1007/s10495-013-0843-5 . 23620435

[42] Benowitz NL, Jacob P 3rd, Slade J, Yu L. Nicotine content of the eclipse nicotine delivery device. Am J Public Health. 1997;87(11):1865–6. 10.2105/ajph.87.11.1865 9366646 PMC1381174

[43] Behan JW, Avramis VI, Yun JP, Louie SG, Mittelman SD. Diet-induced obesity alters vincristine pharmacokinetics in blood and tissues of mice. Pharmacol Res. 2010;61(5):385–90. 10.1016/j.phrs.2010.01.007 20083201 PMC2848885

[44] Shi H, Fan X, Horton A, Haller ST, Kennedy DJ, Schiefer IT, et al The Effect of Electronic-Cigarette Vaping on Cardiac Function and Angiogenesis in Mice. Sci Rep. 2019;9(1):4085 10.1038/s41598-019-40847-5 30858470 PMC6411855

[45] Olfert IM, DeVallance E, Hoskinson H, Branyan KW, Clayton S, Pitzer CR, et al Chronic exposure to electronic cigarettes results in impaired cardiovascular function in mice. Journal of applied physiology. 2018;124(3):573–82. Epub 2017/11/04. 10.1152/japplphysiol.00713.2017 29097631 PMC5899271

[46] Gustavsson P, Jansson C, Hogstedt C. Incidence of myocardial infarction in Swedish chimney sweeps 1991–2005: a prospective cohort study. Occup Environ Med. 2013;70(7):505–7. 10.1136/oemed-2013-101371 . 23596186

[47] Qi D, Young LH. AMPK: energy sensor and survival mechanism in the ischemic heart. Trends Endocrinol Metab. 2015;26(8):422–9. 10.1016/j.tem.2015.05.010 26160707 PMC4697457

[48] Gelinas R, Mailleux F, Dontaine J, Bultot L, Demeulder B, Ginion A, et al AMPK activation counteracts cardiac hypertrophy by reducing O-GlcNAcylation. Nat Commun. 2018;9(1):374 10.1038/s41467-017-02795-4 29371602 PMC5785516

[49] Zhang BB, Zhou G, Li C. AMPK: an emerging drug target for diabetes and the metabolic syndrome. Cell Metab. 2009;9(5):407–16. 10.1016/j.cmet.2009.03.012 . 19416711

[50] Sung MM, Zordoky BN, Bujak AL, Lally JS, Fung D, Young ME, et al AMPK deficiency in cardiac muscle results in dilated cardiomyopathy in the absence of changes in energy metabolism. Cardiovasc Res. 2015;107(2):235–45. 10.1093/cvr/cvv166 26023060 PMC4565988

[51] Zhou P, Ross RA, Pywell CM, Liangpunsakul S, Duffield GE. Disturbances in the murine hepatic circadian clock in alcohol-induced hepatic steatosis. Sci Rep. 2014;4:3725 10.1038/srep03725 24430730 PMC3893658

[52] Hu N, Yang L, Dong M, Ren J, Zhang Y. Deficiency in adiponectin exaggerates cigarette smoking exposure-induced cardiac contractile dysfunction: Role of autophagy. Pharmacol Res. 2015;100:175–89. 10.1016/j.phrs.2015.08.005 . 26276084

[53] Guo S, Yao Q, Ke Z, Chen H, Wu J, Liu C. Resveratrol attenuates high glucose-induced oxidative stress and cardiomyocyte apoptosis through AMPK. Mol Cell Endocrinol. 2015;412:85–94. 10.1016/j.mce.2015.05.034 . 26054749

[54] Zhou X, Sheng Y, Yang R, Kong X. Nicotine promotes cardiomyocyte apoptosis via oxidative stress and altered apoptosis-related gene expression. Cardiology. 2010;115(4):243–50. 10.1159/000301278 . 20339300

[55] Jia F, Wu C, Chen Z, Lu G. AMP-activated protein kinase inhibits homocysteine-induced dysfunction and apoptosis in endothelial progenitor cells. Cardiovasc Drugs Ther. 2011;25(1):21–9. 10.1007/s10557-010-6277-1 . 21258964

[56] Sun Y, Ahokas RA, Bhattacharya SK, Gerling IC, Carbone LD, Weber KT. Oxidative stress in aldosteronism. Cardiovasc Res. 2006;71(2):300–9. 10.1016/j.cardiores.2006.03.007 . 16631148

[57] Sellmann C, Jin CJ, Engstler AJ, De Bandt JP, Bergheim I. Oral citrulline supplementation protects female mice from the development of non-alcoholic fatty liver disease (NAFLD). Eur J Nutr. 2017;56(8):2519–27. Epub 2016/08/09. 10.1007/s00394-016-1287-9 . 27496089

[58] Zhou L, Liu D, Wang Z, Dong H, Xu X, Zhou S. Establishment and Comparison of Juvenile Female Mouse Models of Nonalcoholic Fatty Liver Disease and Nonalcoholic Steatohepatitis. Gastroenterol Res Pract. 2018;2018:8929620 Epub 2018/08/31. 10.1155/2018/8929620 30158971 PMC6109512

[59] Ivey R, Desai M, Green K, Sinha-Hikim I, Friedman TC, Sinha-Hikim AP. Additive effects of nicotine and high-fat diet on hepatocellular apoptosis in mice: involvement of caspase 2 and inducible nitric oxide synthase-mediated intrinsic pathway signaling. Horm Metab Res. 2014;46(8):568–73. 10.1055/s-0034-1375610 24830635 PMC4327908

