# Supplementary material for: Electronic cigarettes cause alteration in cardiac structure and function in diet-induced obese mice
Source: PLoS One. 2020 Oct 1;15(10):e0239671. doi: 10.1371/journal.pone.0239671 (PMC7529198; doi:10.1371/journal.pone.0239671)
Supplement: S1 Raw images — (PPTX) [file pone.0239671.s001.pptx]

## Slide 1
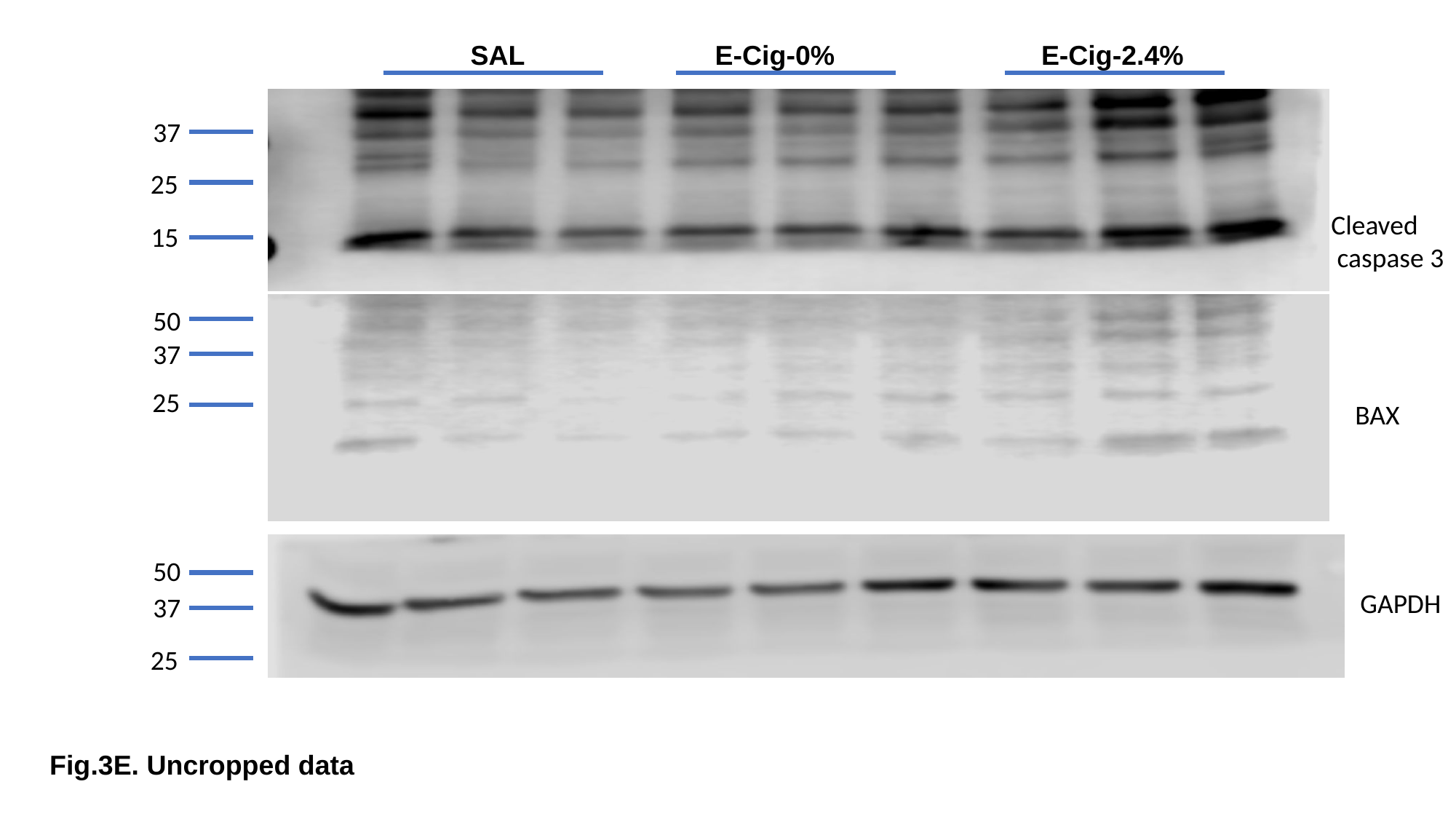

SAL
E-Cig-0%
E-Cig-2.4%
37
25
Cleaved
 caspase 3
15
50
37
25
BAX
50
GAPDH
37
25
Fig.3E. Uncropped data

## Slide 2
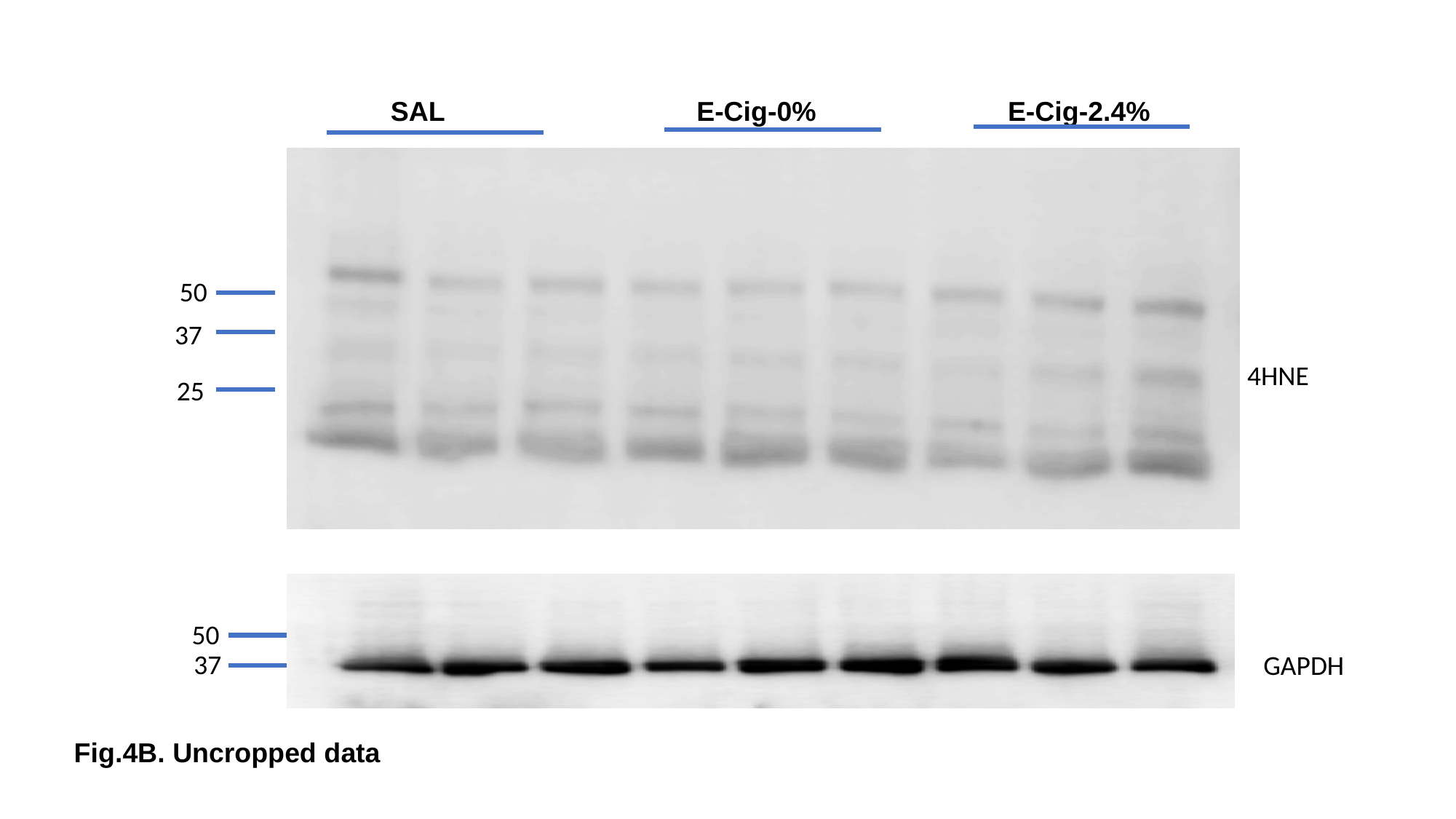

SAL
E-Cig-0%
E-Cig-2.4%
50
37
4HNE
25
50
37
GAPDH
Fig.4B. Uncropped data

## Slide 3
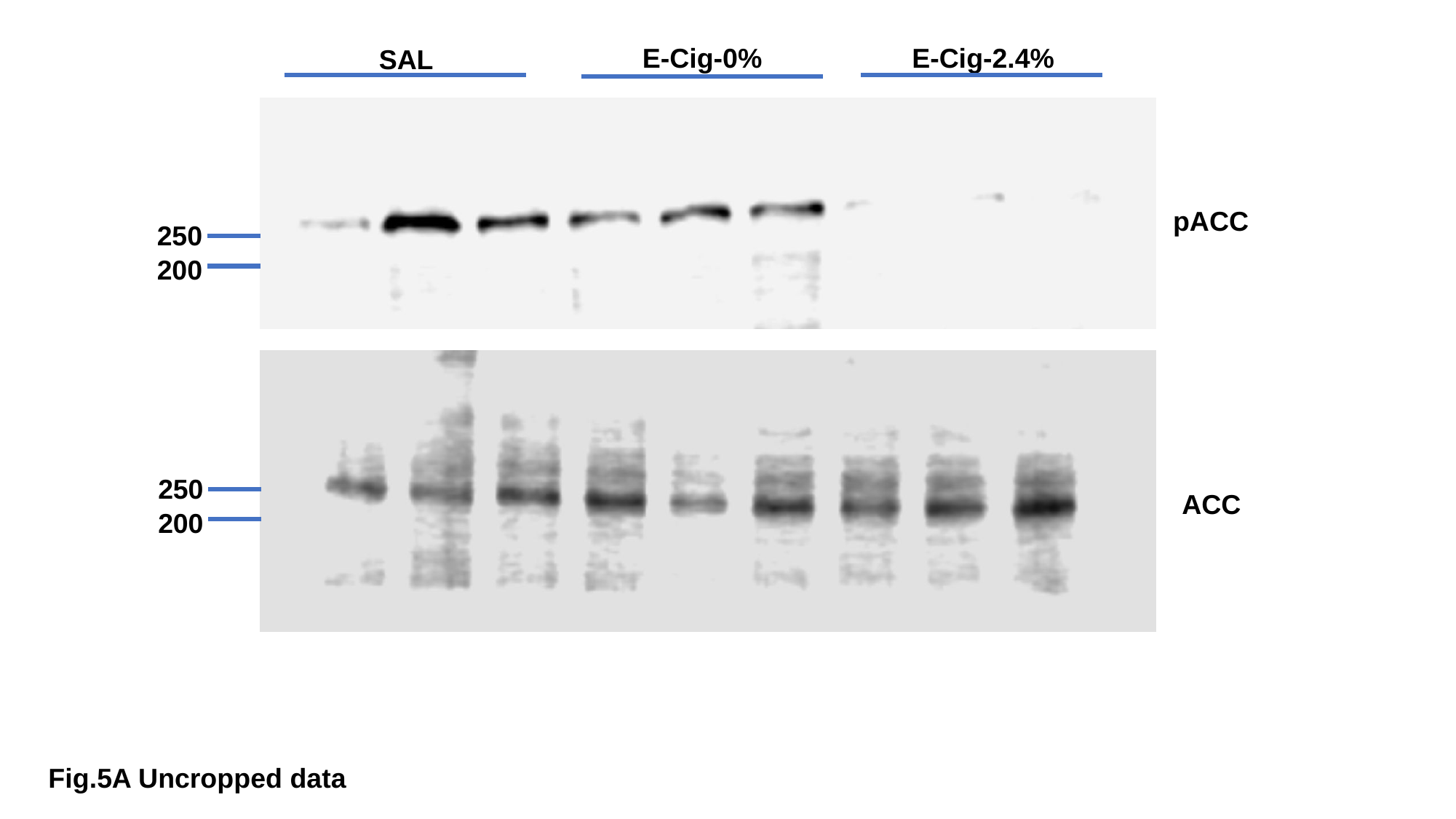

E-Cig-0%
E-Cig-2.4%
SAL
pACC
250
200
250
ACC
200
Fig.5A Uncropped data

## Slide 4
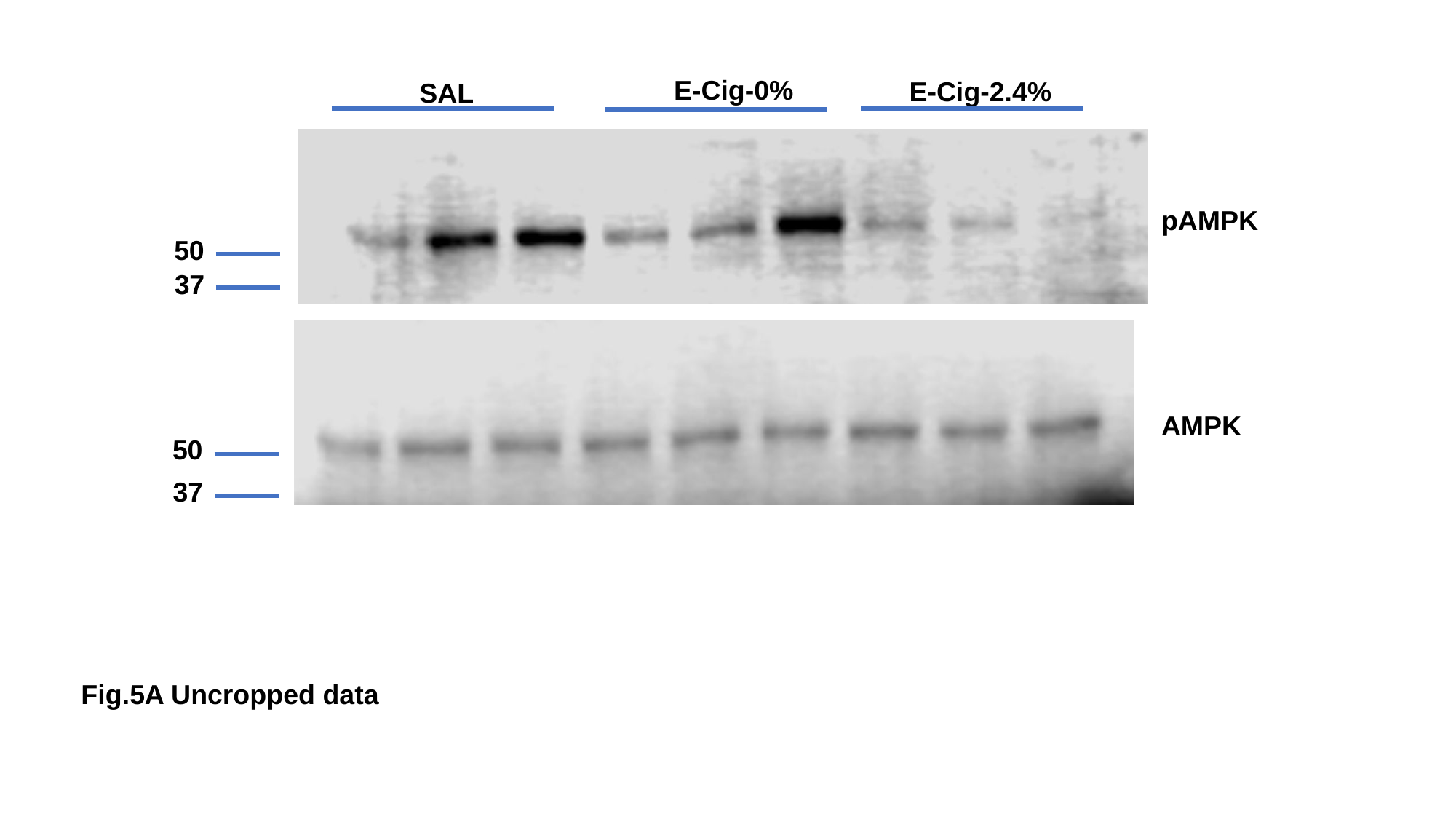

E-Cig-0%
E-Cig-2.4%
SAL
pAMPK
50
37
AMPK
50
37
Fig.5A Uncropped data

## Slide 5
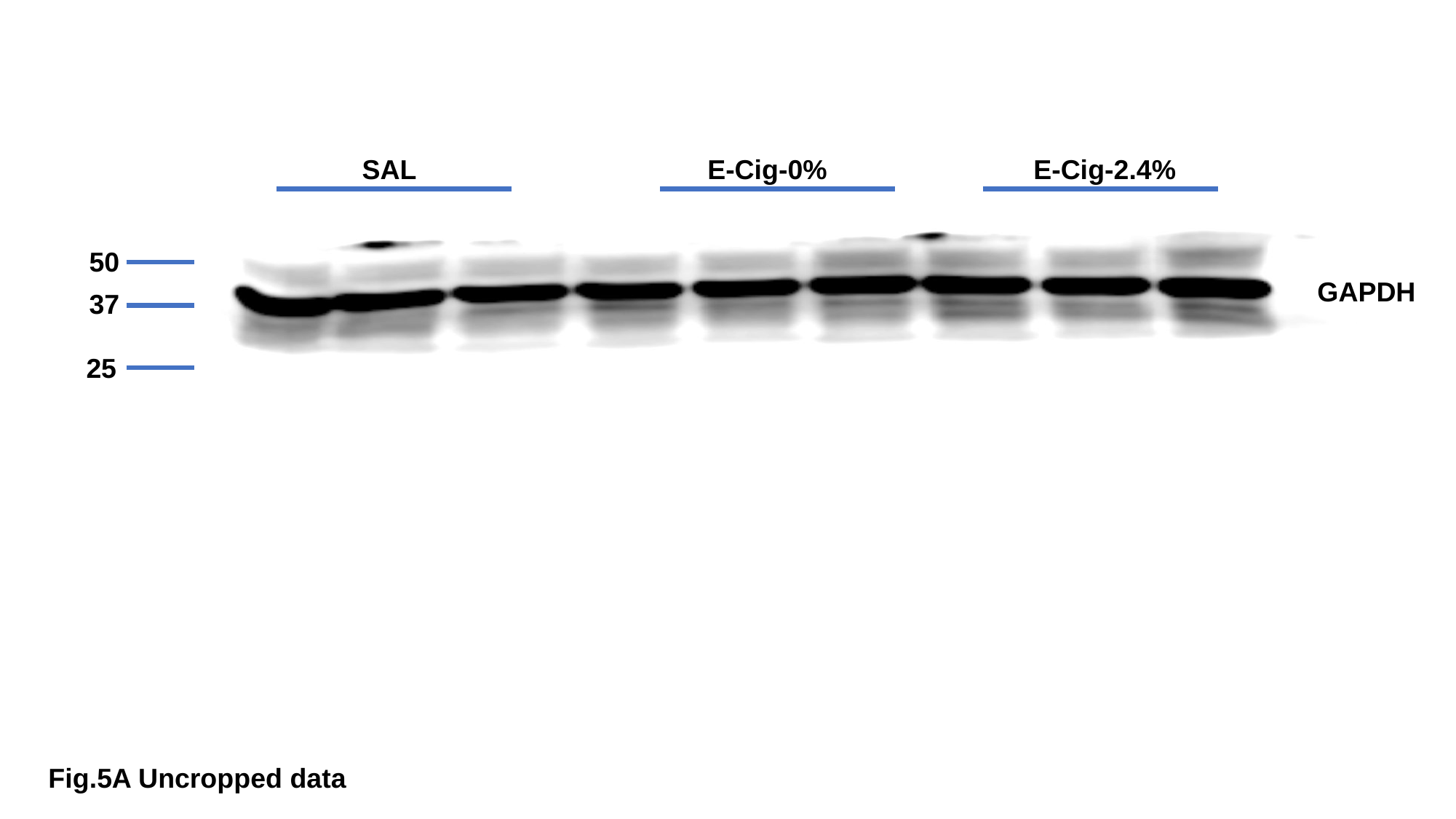

50
GAPDH
37
25
SAL
E-Cig-0%
E-Cig-2.4%
Fig.5A Uncropped data
